# Safety Evaluation of Fungal Pigments for Food Applications

**DOI:** 10.3390/jof7090692

**Published:** 2021-08-26

**Authors:** Rajendran Poorniammal, Somasundaram Prabhu, Laurent Dufossé, Jegatheesh Kannan

**Affiliations:** 1 Department of Natural Resource Management, Horticultural College and Research Institute, Tamil Nadu Agricultural University (TNAU), Periyakulam 625 604, India; kannan.j@tnau.ac.in; 2 Department of Plant Protection, Horticultural College and Research Institute, Tamil Nadu Agricultural University (TNAU), Periyakulam 625 604, India; prabhu.s@tnau.ac.in; 3 Laboratoire de Chimie et Biotechnologie des Produits Naturels (CHEMBIOPRO), Université de La Réunion, ESIROI Agroalimentaire, 15 Avenue René Cassin, F-97400 Sainte-Clotilde, France

**Keywords:** fungal pigments, mycotoxins, safety evaluation, pigment toxicity

## Abstract

Pigments play a major role in many industries. Natural colors are usually much safer when compared to synthetic colors and may even possess some medicinal benefits. Synthetic colors are economical and can easily be produced compared to natural colors. In addition, raw plant materials for natural colors are limited and season dependent. Microorganisms provide an alternative source for natural colors and, among them, fungi provide a wide range of natural colorants that could easily be produced cheaply and with high yield. Along with pigment, some microbial strains are also capable of producing a number of mycotoxins. The commercial use of microbial pigments relies on the safety of colorants. This review provides a toxicity evaluation of pigments from fungal origins for food application.

## 1. Introduction

Due to the global increase in processed food production, the market for fresh food is predicted to grow more in coming years. Modern consumers have become more nutrition and health conscious and have a growing interest in *(i)* where their food comes from and *(ii)* in food additives [1]. Food labeling has increased interest in understanding the physiological needs of the body. Consumers tend to shy away from chemical compounds such as food additives, viz., antioxidants, preservatives and colors. They search for natural additives in natural foods that are safe and good for them. On the other hand, consumers have a growing awareness of natural products where toxicants may also be present [2].

Historically, throughout the world, natural pigments have been used for many purposes. The advent of synthetic dyes reduced the use of natural pigments to a greater extent. Recently, dyes derived from natural sources are gaining importance, since some synthetic dyes have been reported to have carcinogenic effects [3,4]. Natural pigments gained interest due to worldwide concern over the use of eco-friendly and biodegradable materials. Demand for natural colorants, especially yellow and red pigments, israpidly increasing worldwide. Production of safe and natural pigments from natural resources are mainly being focused on in food, textile and pharmaceutical industries, due to the serious environmental and safety problems caused by many artificial synthetic pigments [5].

Many natural pigments are from plant or microorganism-based origins. There are a number of major drawbacks to plant pigments, viz., non-availability throughout the year, pigment stability and solubility. Exploitation of plants on a large scale may lead to a loss of valuable species. For these reasons, the pigments from plant sources are not considered viable. Microorganisms, viz., fungi, bacteria, algae and actinomycetes, are a reliable and readily available alternative source of natural pigments [6,7]. Microorganisms are advantageous over plant for pigments production because of easy and rapid multiplication in a low cost medium, easy processing and growth independent from weather conditions [8].

## 2. Synthetic Food Colors

Color is an important factor as far as food is concerned, as it plays a major role in the taste and perception of food, along with flavor and texture. It is a known fact that consumers will probably reject food that does not look attractive. To make food more appealing to customers, manufacturers add color to retain the food’s natural look, as far as possible. Natural appearance is always preferable to anything that looks unusually colored. Even though many foods can contain added artificial colors, most consumers believe that the color of the food is its natural color.

The first synthetic organic dye, discovered by William Henry Perkin in 1856, was a purplish lilac color named “mauve”. Similar organic aniline dyes were synthesized, representing every color and tint of the rainbow, and these were used for food coloring with little or few tests regarding their safety. Aniline and coaltar-based colors exhibited significant toxicity, which prompted regulators to examine the exact use of synthetic colors in the food industry [9].

In recent years, an astonishing amount of the food we eat is processed. To increase the shelf life and appearance of food, additives and colors are added, which make them unsafe for consumption. On average, processed food accounts for about 70% of the diet of U.S. residents. This includes soft drinks, confectionery, packaged bread, buns, biscuits, cakes, preserved meat products, instant soups, noodles, packaged pizzas, pies and packaged meals. The U.S. consumption of processed food is about forty times more than the diet of Indian residents [10].

### Health Hazards of Synthetic Food Colors

Risk analysis helps to evaluate the adverse effects of synthetic agents in food. In a global context, food colors are of major concern with regard to the possible adverse effects of additives. In the mid-1980s, a possible link between tartrazine and hyperactivity in children was suggested [11].

1. The azo-dye group of colorants consists of bright colors and is widely used in the food industry. Increasing attention on these dyes revealed that they were potential carcinogens, occurring in the intestines’ microbiota, after their azo reduction to carcinogenic metabolites [12]. Even at low levels of ingestion, permitted food colors, viz., ponceau, tartrazine and sunset yellow, provoked allergic reactions in many individuals. Common allergic responses were urticaria, dermatitis, angioedema and the exacerbation of asthmatic symptoms [13]. A symptom of glossitis was reported due to the consumption of a very high level of ponceau 4R in a particular brand of aniseed [14]. Hypertensive children aged between 2 and 14 years were diagnosed with irritability, restlessness and sleep disturbance due to high levels of tartrazine. Ever rising demand for the use of natural colorants has replaced the use of synthetic dyes in food [15].

## 3. Microbial Pigments

Natural pigments obtained from plants, animals and microorganisms are eco-friendly and have usually low or no toxicity [16,17]. The many disadvantages of using plants and animals prevent them from large-scale exploitation [18]. However, advantages of microbial pigments help to utilize their immense potential in various fields [19,20]. Even though the cost of microbial β-carotene production is several times more expensive, it can still compete with synthetic dyes in terms of it being natural and safe [21,22].

**Figure 1:**
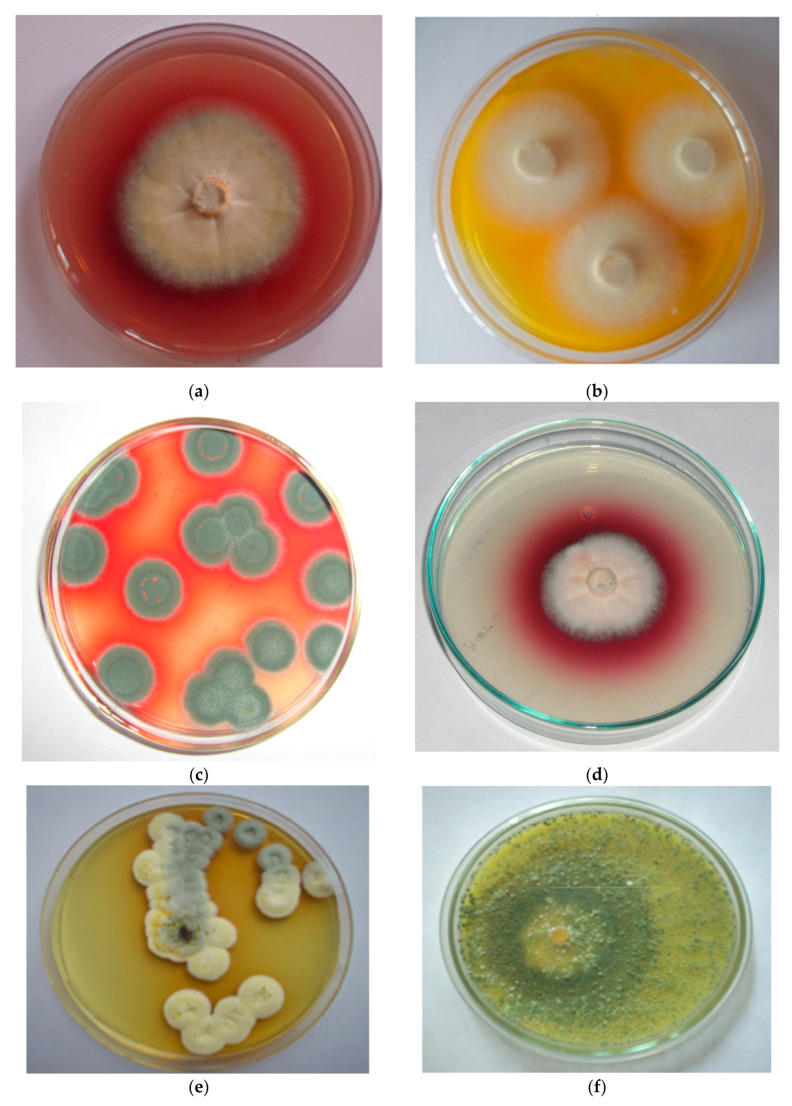
Pigments produced by different fungi: (**a**) *Chaetomium* sp. producing red pigment; (**b**) *Thermomyces* sp. producing yellow pigment; (**c**) *Penicillium purpurogenum* producing red pigment; (**d**) *Fusarium* sp. producing red pigment; (**e**) *Penicillium purpurescens* producing brown pigment (**f**) *Trichoderma* sp. producing yellow pigment.

**Figure 2:**
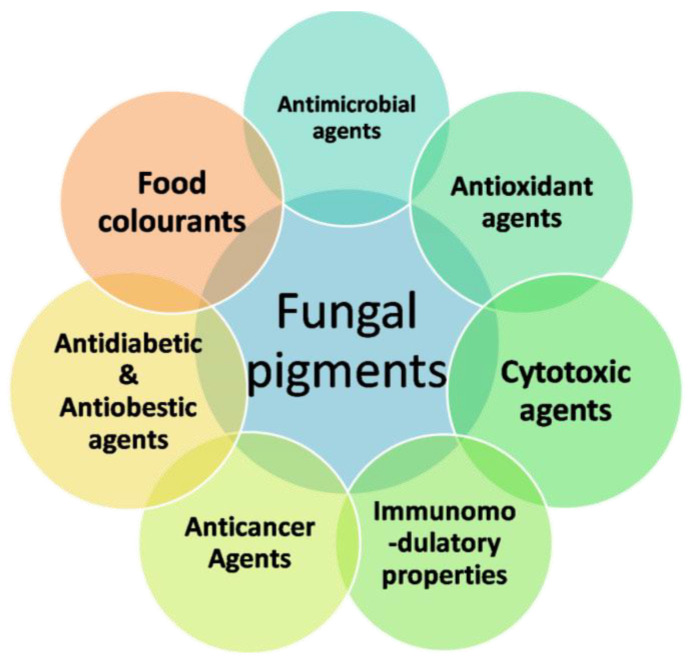
Applications of fungal pigments in the food industry.

Microbial cells that produce color are referred to as microbial pigments producers [23]. They produce a wide range of colors (Figure 1) and are mostly water-soluble [24,25]. Natural pigments are mainly used as color additives or intensifiers; moreover, they are used as antioxidants and antibiotics (Figure 2). Due to indiscriminate use of synthetic colors and contrary reports on the safety of synthetic dyes, there is an important need to identify safe colorants from natural pigments. Microbial pigments have several advantages, viz., yield, cost efficiency, stability and ease of downstream processing compared to pigments from plant or animal origins [26,27].

Among pigment-producing microbes, fungi produce a wide range of water-soluble bio-pigments that have a variety of functions. Pigments extracted from fungi that are isolated from soil have various industrial applications. Filamentous fungi, viz., *Monascus,* *Aspergillus*, *Penicillium*, *Neurospora*, *Eurotium,* *Drechslera* and *Trichoderma* [28,29,30] are potential producers of bio-pigments. The pigments include carotenoids, melanins, flavins, phenazines, quinones, monacins and indigo [31]. Hence, they are the subject of many studies.

Recently, fungal pigments have been used for textile dyes, food colorants, antimicrobial and anticancer applications. They are also natural without having undesirable effects on the environment. Many scientific researchers have proved that pigments from soil fungi are a safer alternative to synthetic colorants, and there is good scope for industrial application [32,33].

## 4. Fungal Pigments and Toxicity Evaluation

Most fungi produce pigments along with mycotoxins. The presence of mycotoxins in pigments restricts the application of pigments as an additive in the food industry [16,34]. The European Union and the United States prohibit the consumption of *Monascus* pigments that are produced along with citrinin toxin, which poses a challenge over its safe use [35]. In short, natural pigments are a potential source of colorants that are eco-friendly, biodegradable, antimicrobial and have antioxidant properties. Apart from food additives, they are also used in cosmetics, pharmaceuticals and drug applications [36] (Table 1).

### 4.1. Aspergillus carbonarius

*Aspergillus carbonarius,* an Ascomycota fungus of the family Aspergillaceae, is capable of producing a yellow-colored pigment in its biomass. It does not produce any antinutrients or mycotoxins [60]. It has been exploited for large-scale production of polygalacturonase and is capable of temperature tolerance by UV irradiation when grown in shake-flask cultures. During the growth phase, a yellow colored pigment is accumulated in its biomass and has the potential to be used as a food colorant [61].

Toxicity studies in both sexes of albino rats at acute and subacute doses of the pigment revealed that feeding of fungal biomass did not show any mortality in rats and there are no significant differences in food intake or organ and body weight. When comparing treated and untreated rats, hematological parameters, serum enzymes lactate dehydrogenase (LDH), alkaline phosphatase (ALP), alanine aminotransferase (ALT or ALAT) and cholesterol assay also remain normal [62].

### 4.2. Blackslea trispora

*Blakesleatrispora* is a Zygomycetes fungus of the order Mucorales, family Choanephoraceae. It is capable of undergoing both sexual and asexual reproduction through the production of zygospores and sporangiospores. The fungus does not produce any toxic compounds; hence, it is of industrial interest as a source of β-carotene for commercial exploitation [63,64]. β-carotene from *B. trispora* was the first authorized microbial food colorant in the European Union. It is efficient and can achieve the highest yield of all trans β-carotene at the expense of other structurally related carotenoids [33]. The process production was improved over a number of years, producing carotenoid contents of up to 20% dry weight [65,66].

The safety assessment of β-carotene, derived from *B. trispora*, has revealed no genotoxicity or subacute toxicity for 4weeks [38,67]. A subchronic toxicity study of 90 days was performed with oral administration of F344.Rats of both sexes showed no adverse effects on their biological systems [39]. β-carotene derived from the *B. trispora* at a 5.0% dietary level, equivalent to 3127 mg/kg/day and 3362 mg/kg/day for male and female rats, caused no adverse effects. The findings revealed that the daily intake of synthetic β-carotene from *B. trispora* by human beings is a negligible toxicological hazard [68].

### 4.3. Fusarium *sp.*

*Fusarium* are Ascomycota fungi that belong to the order Hypocreales, family Nectriaceae. They produce a wide range of fungal pigments that are structurally and functionally diverse. However, among the *Fusarium* sp., *Fusarium graminearum* (red naphthoquinone pigment, *rubrofusarin*) [40], *Fusarium fujikuroi* (orange carotenoids pigment, fusarubin) [43,69] and *Fusarium oxysporum* (red naphthoquinone pigment, bikaverin) [44] are the major pigment-producing fungi. Secondary metabolites from these fungi contain numerous toxic compounds, viz., fumonisins, zearalenone, fusaric acid, fusarins and beauvericins [70].

Toxicity analysis revealed that the red dimeric naphthoquinone pigment from *F. oxysporum*-contaminated products affects human health. Recently, red naphthoquinone pigment has often been reported as a mycotoxin. However, naphthoquinone pigment was not genotoxic according to a DNA synthesis assay. Biotechnological approaches and intelligent screening of the toxic metabolite pathway of the pigment from *Fusarium* sp. will be helpful in producing the pigment for food coloring [41,42].

### 4.4. Monascus *sp.*

*Monascus* sp. are fungi placed under order Eurotiales, family Monascaceae. There are many species in this genus, among which *M. purpureus* (monascorubramine and rubropunctamine) [45], *M. anka* (ankaflavin and monascin) [46] and *M. ruber* (monascorubrinandrubropunctatin) [47] are of greatest significance to the food industry. Traditionally, Monascus pigments were produced on rice using solid-state microbial fermentation. Synonyms for this food product include, Hon-Chi, Hong Qu, Dan Qu, Anka, Ankak rice, Beni-Koji, red koji, red Chinese rice, red yeast rice and red mold rice (RMR). RMR was utilized as a food colorant in traditional Chinese medicine for more than 1000 years [71].

Chinese, as well as other East Asian people, have confirmed the safety of red yeast rice. The European Food Safety Authority (EFSA) and the United States excluded red yeast rice on the list of permissible food additives, due to complex secondary metabolites [72,73]. The toxigenic strain of *Monascus purpureus* is capable of producing nephrotoxic and hepatotoxic mycotoxin citrinin, which limits the wide application of the pigment [74].

For more than a thousand years, pigments produced by *Monascus* sp. were legally used as food colorants in South East Asia, even though they were demonstrated to have physiological effects. There are numerous toxicological data available on this Monascus red pigment.

A genetically modified industrial strain, *M. purpureus* SM001 isolated in China, is capable of producing pigment without citrinin, which is the best *Monascus* pigment producer.This results in the prolonged safety of *Monascus*-related products and their application [75].

### 4.5. Penicillium *sp.*

*Penicillium* are Ascomycota fungi belonging to the order Eurotiales, family Trichocomaceae. They are capable of producing many pigments. *Penicillium* are ubiquitous saprophytic soil fungi, present wherever organic material is available. Several species are capable of producing highly toxic mycotoxins. Some species of the genus *Penicillium* are capable of producing antibiotics, while some other species are used in cheese making; however, pigment production by these fungi is less well known [48,76]. Patents contain information about acute oral toxicity in mice. A 90-day subchronic toxicological study found acute dermal irritation, acute eye irritation, antitumor activity, micronucleus test in mice, AMES test (*Salmonella typhimurium* reverse mutation assay) and an estimation of antibiotic activity, including results of estimation of five mycotoxins [77].

*Penicillium purpurogenum* is capable of producing an azaphilone-like pigment. It secretes a brick red pigment during growth, which generally diffuses into commonly used media. However, violet pigment (PP-V) and orange pigment (PP-O) were also reported by altering culture conditions [78]. The production of pigment from *Penicillium* is more efficient and profitable than any other microorganism. It secretes enzymes and pigments out of the cell and the secreted pigment is water-soluble and relatively stable; thus, it is easily purified [79].

Toxicity studies of *P. purpurogenum* DPUA 1275 on brine shrimp, *Artemia salina,* showed antimicrobial effects and absence of toxicity to go along with pigment production. It also does not produce any known mycotoxins and is nonpathogenic to humans. It is a potential strain for the production of food pigments [80]. Although many species of *Penicillium* are found to produce pigments, only a few toxicological studies have been conducted.

*Penicillium europium*, isolated from forest soil, is capable of producing a pinkish pigment by using longifolene as a sole carbon source. A toxicity study on albino rats revealed that the pigment had no toxic effect on rats. Synthesized pigments from *P. europium* could be used in food, feed and pharmaceutical industries. Apart from the food industry, it could be used for various industrial applications, viz., dyes for textile and non-textile substrates such as paper, leather, paints and cosmetics. Moreover, as it is non cytotoxic, the pigment could be a potential replacement for hazardous synthetic dyes [50,81].

*Penicillium resticulosum* is capable of producing red pigments. An evaluation of the subacute toxicity of oral exposure on the synthesized pigment on adult male and female mice for 28 days, using a pigment dose of up to 500 mg kg^−1^ body weight daily, had no effect on body weight, organ weight, or the activity of lactate dehydrogenase (LDH), alkaline phosphatase (ALP), alanine aminotransferase (ALT or ALAT) enzymes or blood urea nitrogen (BUN) levels. However, mice taking the pigment over 500 mg·kg^−^^1^ body weight daily showed fatty degeneration and mild necrosis of liver cells, indicating that doses under 500 mg·kg^−^^1^ body weight were safe for daily consumption [51,82].

*Penicillium aculeatum* produces a yellow (ankaflavin) pigment under submerged fermentation. Cytotoxicity studies of the pigment interacting with human colon carcinoma cell lines (HCT116) and human prostatic carcinoma cell lines (PC3) exhibited apoptosis and cell cycle inhibition at lower concentrations. An assay of human erythrocytes and human embryonic kidney (HEK-293) cell lines showed the least cytotoxicity atfor highest concentrations tested. Displaying selective cytotoxicity is an important property for an ideal anticancer drug [52].

### 4.6. Talaromyces purpureogenus

*Talaromyces purpureogenus* (basionym: *Penicillium purpureogenum*), is capable of producing yellow and red pigments under submerged fermentation. Pigments from *T. purpureogenus* CFRM02 are non toxic to *Artemia franciscana* (brine shrimp). In a single-dose acute toxicity study, (50, 300, 1000 and 2000 mg/kg body weight) conducted on female Wistar rats, there was no evidence of adverse effects on body weight and mortality after 14 days. Subacute studies (250–1000 mg/kg body weight) showed no significant changes in food intake, body weight gain and relative weight of vital organs after 28 days. Furthermore, a histopathological examination of the liver and kidney was normal. There were no significant changes in serum enzyme activities in the treated and control groups (acute and subacute). Safety efficacy of the pigment from *T. purpureogenus* CFRM02 is suggested for application in food and nutraceuticals [53]. This potential strain has resulted in in-depth studies of some strains of Talaromyces species, viz., *Talaromyces aculeatus*, *T. funiculosum*, *T. pinophilus* and *T. purpurogenum*. They are capable of producing *Monascus*-like polyketide azaphilone pigments, with or without coproducing citrinin or any other known mycotoxins [83].

### 4.7. Thermomyces *sp.*

*Thermomyces* are Ascomycota fungi ofthe order Eurotiales, family Trichocomaceae, and they are capable of producing a yellow pigment. They are thermophilic and hemicellulose degraders. One strain was isolated from soil in Kodaikanal, Dindugul District, Tamil Nadu, India. The yellow pigment can scavenge reactive oxygen species (ROS) induced by chemicals and UV rays and reduce the DNA damage by their antioxidant capabilities [84]. The bright pigmentation varies from yellow to red, depending on the growth, temperature, age and substrate [85].Food and beverages fortified by the yellow pigment recorded high antioxidant properties, antimicrobial properties and color stability [86].

**Figure 3:**
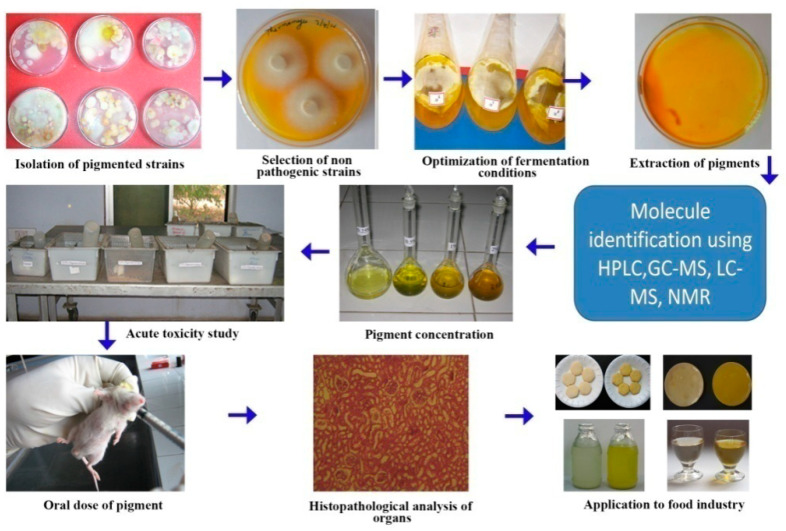
Toxicity evaluation of *Thermomyces* sp. pigment using an animal model.

Toxicological studies on the *Thermomyces* pigment using albino mice that orally ingested *Thermomyces* sp. pigment for 28 days showed no alterations in red blood cells, white blood cells, haemoglobin, organ weight orhistopathology of the liver or kidneys [54] (Figure 3). Apart from pigmentation, in vivo antioxidant activity was also observed [87,88]. Therefore, pigments from *Thermomyces* sp. could be safe for the food industry.

### 4.8. Trichoderma viride

*Trichoderma viride* is an Ascomycota fungus belonging to the order Hypocreales, family Hypocreaceae. It is capable of producing a brown colored pigment, identified as furfural (only one study and this should be confirmed) [89]. It is mostly used as a biological control agent against soil-borne plant pathogenic fungi. It is also capable of synthesizing emodin, a yellow pigment. *T. polysporum* is the only fungi capable of producing emodin in culture media. A phytotoxicity assay proved the nontoxic nature of *T. viride* on the germination of *Phaseolus aureus* Roxb. The pigment, i.e., furfural, produced by *Trichoderma viride*, has possible wide applications in various industries [89].

### 4.9. Scytalidium cuboideum

*Scytalidium cuboideum*, an Ascomycota fungus belonging to the order Helotiales, family Chaetomiaceae, is isolated from wood and is capable of coloring wood. It produces a red pigment, identified as xylindein.

Toxicity studies of the red pigment in zebrafish revealed a relatively high LD50 value, making it unlikely to affect humans. Pure solidified pigments from *S. cuboideum* did not demonstrate toxicity. However, significant mortality was associated with impure fungal metabolites containing pigments. Hence, xylindein is considered as an environmentally safe pigmentation for future applications [55,90].

### 4.10. Neurospora crassa

*Neurospora crassa* is a type of red bread mold of the phylum Ascomycota in the order Sordariales, family Sordariaceae. It is a genetically and biochemically well-studied eukaryotic microorganism [91,92]. It is capable of producing polyketide and carotenoid fungal pigments that are yellow to orange-red pigments widely used as food colorants. Research on *Neurospora* sp. has generally recognized it as safe after more than two centuries, with no record of mycotoxin production. For the production of ethanol, biomass and pigments, it can be grown rapidly on industrial residuals and lignocelluloses.

### 4.11. Other Fungal Pigments

The Cordycipitaceae family have promising pigment-producing genera such as *Torrubiella*, *Cordyceps, Beauveria*, *Hyperdermium* and *Lecanicillium*. *Beauveria bassiana* produces tenellin and *Beauveria brongniartii* produces bassianin. *Beauveria bassiana* produces pyridovericin, blood-red dibenzoquinone and pyridomacrolidin, along with mycotoxin. *Torubiella* produces torrubiellones and *Lecanicillium aphanocladii* produces a pigment, along with oosporein [93]. This molecule has a wide range of bioactivities from antifungal, antimicrobial and phytotoxic effects to growth inhibition in plants. Additionally, kidney damage and even death were noticed in poultry exposed to oosporein.

## 5. Yeast

Yeast synthesizes a variety of commercially important carotenoids, viz., carotene, torulene, torularhodin and astaxanthin. Genera *Rhodotorula*, *Sporobolomyces* and *Phaffia* are potential sources of pigments. Carotenoids from yeast are used in pharmaceutical, chemical, food and feed industries. They are also an important precursor for vitamin A synthesis. Apart from coloring, it also has antioxidant and possible tumor-inhibiting properties [94,95].

### 5.1. Rhodotorula *sp.*

Rhodotorula a pigment-producing yeasts from division Basidiomycota are capable of synthesizing carotenoid pigments. Other species such as *R. gracilis*, *R. rubra* and *R. graminis* are also capable of producing pigments. The main compounds produced by *Rhodotorula glutinis* (red yeasts) are torulene and torularhodin, with a minute quantity of β-carotene [96,97].

A toxicological evaluation of *R. gracilis* CFR-1, using acute doses in freeze-dried form at 0.5–6.0 g/kg of body weight (*w*/*w*), did not show any toxic symptoms or mortality in adult rats. Dietary intake at low concentrations of 0.1–2.0% level (*w*/*w*) for 14 weeks did not induce any significant change in food intake and body weight gain in experimental rats compared to control animals [56,57].

Toxicity studies of β-carotene, torulene and torularhodin produced by *R. glutinis* conducted on rats demonstrated that they can be used as safe food additives. Dry powdered *R. glutinis* NCIM 3353 yeast biomass was added to the fodder of rats. The rats had protective effects against precancerous lesions of the liver induced by N-nitrosodimethylamine [98].

### 5.2. Yarrowia lipolytica

*Yarrowia lipolytica* is an Ascomycetous yeast and the only described species of the genus Yarrowia. It is widespread in nature, has many industrial uses and is important in the food industry and medical field. It is used as an alternative source of β-carotene. The major constituents of the carotenoids include all-trans-β-carotene with small amounts of 9-cis β-carotene, 15-cis β-carotene, 13-cis β- carotene and others [99].

Toxicity studies of β-carotene from *Y. lipolytica* in genotoxicity models and a standard subchronic rat study revealed no significant difference, compared with commercial products. An extracellular nontoxic pyomelanin pigment from *Y. lipolytica* was found to have antioxidant and noncytotoxic properties for two mammalian cell lines, viz., mouse fibroblast (NIH3T3) and human keratinocytes (HaCaT). Purified pyomelanin has a significant sun protection factor (SPF) value, highlighting its potential as a UVfilter in cosmetic preparations. Biomass of this yeast was defined as a safe novel food by the European Food Safety Authority [100].

## 6. Conclusions

In recent decades, natural pigments have been extensively used as colorants in food, pharmaceutical, cosmetic and textile industries. Several fungal strains are known for pigment production, while many fungi have not been systematically explored for their pigment-producing capability. Therefore, there is a need to explore novel and safe pigments using appropriate tools and techniques. Many pigments, including those with antibiotic-like properties, need to be studied for selective toxicity so that they can be produced commercially for human use. Fungal pigments open many new avenues in the production of textiles for medical use. This provides an extensive area of exploration to identify natural, eco-friendly pigments for diverse applications to satisfy public demand. In addition, biotechnological approaches help to produce pigments on a large scale with low cost, high yield and easy extraction without mycotoxins. The manufacture of fungal pigments has taken a big step to promote eco-friendly pigments. A literature search reveals the application of fungal pigments in the food and health care industries. These fungal pigments need to pass toxicity tests, quality tests and regulatory approval before their final entry into the market as food colorants or as drugs. Moreover, toxicology testing for most of the fungal pigments was not available. Another major impediment is that the funding required to carry out the necessary safety studies on such food additives is not available. For the above pigments to be feasible, testing is imperative. Fungal pigments could be a boon to the food industry.

## Figures and Tables

**Table 1:** Important fungal pigments and their safety evaluation.

Fungus	Pigment(s)	Color	Mycotoxin(s)	Safety Evaluation	Biological Activity	Reference(s)
*Aspergillus carbonarius*	Melanins	Yellow	Not described/not found up to now	Subacute toxicity study	Antioxidant	[37]
*Blackeslea trispora*	ß-carotene	Red-orange	Aflatoxin Mycotoxin	Genotoxicity and subacute toxicity study	Antioxidant, anticancer, suppression of cholesterol synthesis	[38,39]
*Fusarium graminearum*	Rubrofusarin	Red	Fumonisins, Zearalenone, Fusaric Acid, Fusarins and Beauvericins	Cytotoxic in colon cells	Antimicrobial, antiallergic, phytotoxicic	[40,41,42]
*Fusarium fujikuroi*	Fusarubin	Orange	Fumonisins, Zearalenone, Fusaric Acid, Fusarins and Beauvericins	Cytotoxic against leukemia cells	Anticancer, antimicrobial	[42,43]
*Fusarium oxysporum*	Bikaverin	Red	Fumonisins, Zearalenone, Fusaric Acid, Fusarins and Beauvericins	Cytotoxic against tumour cells, apoptosis suppressor	Antimicrobial, antitumour	[44]
*Monascus purpureus*	Monascorubramine rubropunctamine	Red	Citrinin	Acute oral toxicity	Antihypertensive metabolite	[45]
*Monascus anka*	Ankaflavin and Monascin	Yellow	No coproduction of toxin	Acute oral toxicity	Antibacterial, antitumor and immunosuppressive	[46]
*Monascus ruber*	Monascorubrin and rubropunctatin	Orange-red	No coproduction of toxin	Oral toxicity	Anti-inflammatory, anticancer andantihyperlipidemic activities	[47]
*Penicillium purpurogenum*	Azaphilone	Brick red pigment	No coproduction of toxin	Brine shrimp *Artemia salina* study	Pharmaceutical and food industry	[3,48,49]
	Purpurogenone	Yellow-orange				
	Mitorubrino	Orange-red				
	Mitorubrin	Yellow				
*Penicillium europium*	Benzoquinone	Pinkish red	Nontoxic	Subacute toxicity study	Antimicrobial	[50]
*Penicillium resticulosum*	Not described/not found up to now	Red	Nontoxic	Subacute toxicity study	Antimicrobial	[51]
*Penicillium aculeatum*	Ankaflavin	Yellow	Nontoxic, selective toxicity in cancer cells	Cytotoxicity study	Antimicrobial	[52]
*Talaromycespurpureogenus*	Purpuride, monascorubrin, purpurquinone-A, ankaflavin,	Yellow and red	Nontoxic	Subacute toxicity study	Antioxidant	[53]
*Thermomyces* sp.	Napthoquinone	Yellow	Nontoxic	Subacute toxicity study	Antioxidant, antimicrobial and food industry	[54]
*Trichoderma viride*	Emodin Viridol	Brown Yellow	Nonphytotoxic	Phytotoxicity assay	Antimicrobial	[28]
*Scytalidium cuboideum*	Xylindein	Red	Nontoxic	Zebrafish toxicity study	UV resistant	[55]
*Rhodotorula glutinis*	β-carotene, torulene and torularhodin	Red and orange	Nontoxic	Standard subchronic toxicity study	Antioxidant, Antimicrobial Food and feed additive	[56]
*Rhodotorula gracilis*	β-carotene, torulene and torularhodin	Red and orange	Nontoxic	Standard subchronic toxicity study	Antimicrobial	[57,58]
*Yarrowia lipolytica*	β-carotene	Brown	Nontoxic	Genotoxicity models and a standard subchronic toxicity study	Antimicrobial	[59]

## Data Availability

Not applicable.
